# Metabolic Syndrome Increases Risk of Readmission and Complications in Operative Fixation of Pilon Fractures

**DOI:** 10.7759/cureus.41283

**Published:** 2023-07-02

**Authors:** Zachary A Panton, Rachel Ranson, Malcolm DeBaun, Nishant Suneja, Christian Pean, Mark Fleming

**Affiliations:** 1 Orthopaedic Surgery, Geisel School of Medicine at Dartmouth College, Hanover, USA; 2 Orthopaedic Surgery, George Washington University Hospital, Washington D.C., USA; 3 Orthopaedic Surgery, Duke University Hospital, Durham, USA; 4 Orthopaedic Surgery, Brigham and Women's Hospital, Boston, USA; 5 Orthopaedic Surgery, Massachusetts General Hospital, Boston, USA

**Keywords:** value based care, patient outcomes, orthopaedic trauma, pilon fracture, metabolic syndrome (mets)

## Abstract

Background

Studies demonstrate that metabolic syndrome (MetS) negatively impacts surgical outcomes. This study sought to identify how metabolic syndrome affects outcomes after open reduction and internal fixation (ORIF) of traumatic pilon fractures.

Methods

Patients who underwent ORIF for pilon fractures from 2012 to 2019 were identified in the American College of Surgeons National Surgical Quality Improvement Program database. Patients with MetS were compared to non-MetS patients for rates of adverse events, prolonged stay, readmission, discharge location, and operative time in the 30-day postoperative period. All statistical analyses were conducted using SPSS version 26.0 (IBM Corp., Armonk, NY, USA). Paired student t-tests were used to assess continuous variables. Pearson’s Chi-square and odds ratios were used for categorical variables.

Results

A total of 1,915 patients met this study’s inclusion criteria, and 127 MetS patients were identified in the cohort. The MetS cohort was older (62.7 vs 49.5 years old, p-value <0.01), with a greater proportion of female patients (59.1% vs 50.2%, p=0.054). MetS patients experienced significantly higher rates of infectious complications (7.9% vs 3.9% OR 2.75 (CI 1.36-5.53), p=0.008), major adverse events (11% vs 4.3%, OR 2.79 (CI 1.53-5.09) p=0.002), and readmissions. MetS patients also had longer lengths of stay (7 days vs 3.8 days, p-value<0.001), and were more likely to be discharged to a non-home location (51.2% vs 19.5%, p-value<0.01, OR 4.32 (CI=3.0-6.24) p<0.001).

Conclusion

Patients with MetS have an increased risk of 30-day major complications, infection, readmissions, discharge to a non-home location, and prolonged operative time, and therefore warrant additional consideration for perioperative monitoring.

## Introduction

Metabolic syndrome (MetS) is a disorder that exists as a combination of hypertension, hyperlipidemia, type 2 diabetes, truncal obesity, and hypertriglyceridemia [1]. Although the specific diagnostic criterion for MetS continues to be debated, many agree that MetS prevalence will continue to rise in global settings [1,2]. Some studies estimate that MetS prevalence is as high as 33% within the United States and is expanding [2]. Given the rapid growth of the MetS patient population, this condition will have a larger role in dictating contemporary healthcare policy, preventative care measures, and treatment protocols both domestically and internationally.

Studies have shown patients with MetS experience increased morbidity and mortality when compared to other patients suffering from various medical conditions and undergoing common surgical procedures, such as thyroidectomy or emergency appendectomy [3-6]. Furthermore, there is an emerging branch of literature linking MetS to worsened surgical outcomes within orthopaedics and several studies demonstrate patients with MetS to have increased readmissions and complications in the 30-day period following open reduction and internal fixation (ORIF) for ankle fracture as well as increased rates of wound complication after hip arthroplasty [7,8]. Components of MetS, such as type 2 diabetes, are known to increase fracture and infection risk and to adversely affect orthopaedic surgical outcomes through various pathways [6,9-12]. Nonetheless, little is known about the relationships between the constellation of metabolic disorders comprising MetS, surgical outcomes and risk, and postoperative complication rates within the field of orthopaedics, particularly in relation to pilon fractures.

Pilon fractures are defined as any fracture that involves the distal articular surface of the tibia [13]. These fractures are well known to be associated with higher rates of complications such as infection, malunion, and non-union [13,14]. Additionally, this fracture type often presents with complex or comminuted fracture patterns, further complicating care [13,14].

As shifts to value-based care occur, it is critical to properly risk-stratify patients to develop policy and prevent patient complications when possible, particularly in the setting of a fracture pattern fraught with complication in a complex patient population. This study sought to identify potential risk factors in patients with MetS that affect rates of adverse events, length of stay, and readmission in the 30-day postoperative period after open reduction and internal fixation (ORIF) of pilon fractures. We predict that given the complex relationships between diabetes, hypertension, hyperlipidemia, central adiposity, and bone health, patients with MetS will experience increased complication rates after operative treatment of pilon fracture. Additionally, this study aspires to identify and highlight specific areas where strategic intervention could better address the clinical demands of MetS patients suffering from traumatic pilon injury.

This work was presented at the Society of Military Orthopaedic Surgeons (SOMOS) Annual Meeting from December 12-16, 2022, and the Medical Student Orthopaedic Society (MSOS) 2023 Symposium on April 23, 2023.

## Materials and methods

Patients who underwent inpatient ORIF for pilon fractures from 2012 to 2019 were retrospectively identified in the American College of Surgeons National Surgical Quality Improvement Program (ACS-NSQIP) database using Current Procedural Terminology (CPT) codes 27827 and 27828. Inclusion criteria were diagnosis of pilon fracture by CPT code and adult age (18+). Demographic variables such as age, race, and sex were collected. Patients were stratified into the MetS and non-MetS cohorts using diagnosis information provided by the ACS-NSQIP dataset. The diagnosis of MetS was made based on criteria from the NSQIP database literature. Patients with concomitant diagnoses of hypertension requiring medication, BMI>30, and diabetes (either insulin-dependent or requiring medications) were considered to have metabolic syndrome [15]. Patients with MetS were compared to other patients for rates of adverse events, such as infection, discharge location, length of stay, and readmission in the 30-day postoperative period. Major adverse events were considered the occurrence of deep surgical site infection, organ-space infection, postoperative sepsis or septic shock, wound dehiscence, mortality, respiratory complications (pneumonia, failure to wean from the ventilator, unplanned reintubation), pulmonary embolism, cardiovascular complications (myocardial infarction, cardiac arrest, stroke), acute renal failure and return to the operating room. Infectious complications included deep surgical site infection, organ-space infection, postoperative sepsis or septic shock, or urinary tract infection.

All statistical analyses were conducted using SPSS version 26.0 (IBM Corp., Armonk, NY, USA). Paired student t-tests were used to assess continuous variables. Pearson’s Chi-square and odds ratios were used for categorical variables.

## Results

A total of 1,915 patients met the inclusion criteria for this study. In total, 127 MetS patients were identified in the cohort. Patients with MetS were older (62.7 vs 49.5 years old, p-value <0.01) and had a greater proportion of female patients (59.1% vs 50.2%, p=0.054). Patients with MetS also had a higher risk of several comorbidities (Table 1).

**Table 1:** Demographic variables for patients operatively treated for pilon fractures with and without metabolic syndrome *=p-value<0.01, statistically significant; MetS=Metabolic Syndrome, CI=Confidence Interval, COPD=Chronic obstructive pulmonary disease.

	General Cohort N=1788 (N, %)	MetS Patients N=127 (N, %)	OR (95% CI), p-Value
Gender*			
Female	897 (50.2)	75 (59.1)	1.21 (0.98-1.51)
Age* (Mean)	49.5	62.7	P<0.001
Race*			
Hispanic	178 (10.0)	11 (9.3)	0.86 (0.45-1.62)
Asian	23 (1.3)	1 (1.2)	0.61 (0.08-4.55)
Black	176 (9.8)	10 (9.2)	0.78 (0.40-1.52)
White*	1103 (61.7)	95 (57.6)	0.54 (0.35-0.82)
Bleeding Disorder*			
Yes	75 (4.2)	11 (8.7)	2.17 (1.12-4.19)
Smoker			
Yes	521 (29.1)	28 (22.0)	0.69 (0.45-1.06)
History of COPD*			
Yes	69 (3.9)	18 (14.2)	4.11 (2.37-7.16)
History of Cancer			
Yes	9 (0.5)	2 (1.6)	3.16 (0.67-14.80)
Acute Renal Disease			
Yes	2 (0.1)	1 (0.8)	7.09 (0.64-78.7)
Renal Dialysis*			
Yes	12 (0.7)	6 (4.7)	7.34 (2.71-19.89)

Patients with MetS had a significantly higher rate of infectious complications (7.9% vs 3.0% OR 2.75 (CI 1.36-5.53), p=0.008), major adverse events (11% vs 4.3%, OR 2.79 (CI 1.53-5.09) p=0.002), and readmission than other patients undergoing operative fixation for pilon fractures (Table 2). Patients with MetS had a longer length of stay (7.1 days vs 3.8 days, p-value<0.001), and were more likely to be discharged to a non-home location (51.2% vs 19.5%, p-value<0.01, OR 4.32 (CI=3.0-6.24) p<0.001). Additionally, patients with MetS had statistically significantly longer operative times. Surgeries for patients with MetS spanned a mean of 144 minutes +/- 81 minutes, compared to a mean of 118 minutes +/- 67 minutes for the non-MetS cohort (p<0.01).

**Table 2:** Adverse event comparisons of patients with and without metabolic syndrome *=p-value<0.01, statistically significant. MetS=Metabolic Syndrome, CI=Confidence Interval

	General Cohort N=1788 (N, %)	MetS Patients N=127 (N, %)	Odds Ratio (95% CI)	P-Value
Any Adverse Event*	137 (7.7)	25 (19.7)	2.95 (1.85-4.73)	<0.01
Major Adverse Event*	76 (4.3)	14 (11.0)	2.79 (1.53-5.09)	0.002
Infectious Complications*	54 (3.0)	10 (7.9)	2.75 (1.36-5.53)	0.008
Mortality	8 (0.4)	2 (1.6)	3.56 (0.75-16.9)	0.14
Discharge not Home*	349 (19.5)	65 (51.2)	4.32 (3.00-6.24)	<0.001
Readmission*	81 (4.5)	12 (9.4)	2.20 (1.17-4.15)	0.03
Length of Stay*	3.8 +/- 7 days	7.1 +/- 9 days		<0.01
Operative Time*	118 +/- 67 minutes	144 +/- 81 minutes		<0.01

## Discussion

Pilon fractures remain a very complicated fracture pattern for orthopaedic surgeons, and they are associated with high rates of complication and morbidity [13-14,16]. This study demonstrates that MetS is a strong risk factor for major complication after ORIF for pilon fracture, as 7.0% of MetS patients experienced major complication compared to 2.7% of non-MetS patients (OR 2.68). Post-surgical complications are estimated to increase hospital costs by 1.5x, and rates of increased length of stay and discharge to place other than home are also linked with increased major complication risk [17]. In this study, patients with MetS spent over 3.5 additional days in care settings when compared to non-MetS patients and were approximately 1.5x as likely to not discharge home. Orthopaedic risk mitigation in this complication-prone population is essential for overall cost reduction, improved patient outcomes, and decreased morbidity [17].

The literature suggests that infection is a devastating complication for orthopaedic patients after ORIF, and this study’s results have implications for evaluating postoperative infection risk for MetS patients suffering from pilon fractures. Patients with MetS in this cohort had a 6.3% rate of infection versus 3% of patients without MetS (OR 2.16). Additionally, Molina et al., while investigating risk factors affecting deep infection rates in pilon fracture fixation, found that patients with hypertension were 2.34 times more likely to develop deep infection after surgical correction of their fracture [14]. Wukich et al. also found that patients with more severe cases of diabetes were 3.7 times more likely to develop an infection after operative fixation of ankle fracture than those with less severe cases [12]. Our study is unique, however, in that it analyzes infection in MetS, which encompasses diabetes, hypertension, and other metabolic derangements. Our data confirm that infection risk is a factor that orthopaedic surgeons must continually mitigate when caring for this patient population.

This study illustrates that patients with MetS are more likely to be readmitted after pilon fracture in comparison to other patients. Patients with MetS had a 9.1% readmission rate after discharge compared to a 3.7% readmission rate in non-MetS patients (OR 2.62). Our data reaffirm findings from recent literature that show MetS to be a risk factor for increasing 30-day readmission rate after ankle fracture [8]. These findings further establish that patients with MetS require careful perioperative management to reduce readmission. Orthopaedic surgeons must be aware of the potential risks of discharging these patients without adequate post-discharge plans and timely outpatient follow-up.

This study noted that operative time was significantly longer in the MetS cohort versus the non-MetS cohort. The Xie et al. study, which analyzed the impact of MetS on post-operative outcomes after ankle fracture, observed differences that approached statistical significance (p = .054), but ultimately fell short [8]. Conversely, Cheng et al. found no significant differences (p = 0.70) in operative time for MetS and non-MetS patients undergoing total hip arthroplasty [7]. This finding is subject to scrutiny, given that fracture classification is not an available variable in the ACS-NSQIP study. Patients with MetS are more likely to be obese which could contribute to operative time. Collins et al. describe many potential pathways in which chronic, low-level inflammation spurred by obesity could contribute to poor bone and tendon quality, and therefore create additional challenge in fixation [18]. For our study, no insight on the quality of bone for fixation or whether these patients had inherently more complex injuries is available. These inconsistent findings require further investigation to definitively elucidate whether operative time is a targetable risk factor to improve postoperative outcomes in the MetS cohort.

Interestingly, despite the increased rate of complications witnessed amongst the MetS cohort in our study, there was no statistically significant difference in rates of mortality between the MetS and non-MetS cohorts. Cichos et al. studied the effects of MetS on outcomes after definitive treatment of hip fracture and reported similar results about mortality [19]. Conceivably, components of MetS, such as type-2 diabetes and obesity, would have negative impacts on mortality, given what is known about the pathophysiology of atherosclerosis, coronary artery disease, and acute coronary syndrome [20]. This contradictory information is consistent with the “obesity paradox”, the phenomenon in which increased adiposity and body mass index (BMI) is associated with protective effects over mortality [21-22]. Nonetheless, because of the lack of consensus for whether higher BMI is truly protective over mortality, our study’s conclusion about mortality warrants additional investigation.

MetS is becoming increasingly relevant to public health professionals as well as orthopaedic surgeons because of the MetS population’s expanding size and the expected effects from increased stressors present during the COVID-19 pandemic [1,23-24]. Patients with MetS have been shown to have increased susceptibility to the COVID-19 virus, and the metabolic health of these patients is expected to suffer significantly during the pandemic given the psychosocial and socioeconomic hardships limiting access to resources such as healthy foods, community centers, and fitness centers [1,24].

This study also has major implications for informing value-based care. The transition to bundled payments from fee-for-service incentive structures may place certain care systems at disadvantages. For example, systems that treat patient populations suffering from high energy traumas or high rates of postoperative complication have increased financial pressure in providing comprehensive care under bundled payment constraints [25-27]. Similarly, given recent advances to create performance-based reimbursement systems based on quality from CMS and private payers, it is vital to have information that informs how specific comorbidities impact postoperative outcomes and quality measures after pilon fracture [28]. This study can further inform policy for designing reimbursement models, given that MetS patients seemingly have increased susceptibility to postoperative complications after ORIF of pilon fracture. Similarly, the results suggest improving overall access to primary care to alleviate the subsequent financial and systemic burdens of comorbidities on postoperative complication rates.

While our study provides important evidence supporting policy and care delivery changes for how pilon fractures are addressed in patients with MetS, there are limitations to our study. Firstly, this study analyzes the effects of MetS that occur primarily in the 30-day period postoperatively. It is possible that the trends seen from this study of MetS and pilon fracture are not reflective of relationships and outcomes in longer-time parameters. Future study should analyze risk factors and complication rates over longer periods of time to elucidate important factors to consider for both short-term and long-term management of pilon fracture in MetS, after ORIF. Furthermore, future studies should aim to further qualify how the degree to which MetS is controlled affects complication rate. Diabetes is well-studied to be a risk factor for complication in many surgical settings and is often used in risk stratification tools, such as the Charlson Comorbidity Index; furthermore, this data would provide more context surrounding how adequate preventative and primary care could better control MetS and ameliorate infection risk in this patient population.

## Conclusions

In conclusion, our study results emphasize the importance of future study of MetS to better characterize the relationship of this syndrome to bone health and surgical outcomes. A better understanding of MetS may expose modifiable factors that can help to mitigate complication risks in this cohort. The results of this study support the implementation of proactive preventative health measures and societal health policies that address the prevalence of MetS. While experts continue to explore the specific relationships and pathophysiology of MetS, our evidence reaffirms that these patients have poor surgical outcomes after surgery for pilon fractures. Orthopaedic surgeons should continue to care for this patient population with caution and recognize that MetS is an increasingly common comorbidity, associated with higher rates of infection, 30-day readmission, and major complication after surgery.
